# The impact of aging on neutrophil functions and the contribution to periodontitis

**DOI:** 10.1038/s41368-024-00332-w

**Published:** 2025-01-16

**Authors:** Zi Wang, Anish Saxena, Wenbo Yan, Silvia M. Uriarte, Rafael Siqueira, Xin Li

**Affiliations:** 1 Department of Plastic Surgery, Maxillofacial & Oral Health, University of Virginia School of Medicine, Charlottesville, VA USA; 2 Molecular Pathobiology Department, New York University College of Dentistry, New York, NY USA; 3 Department of Oral Immunology and Infectious Diseases, University of Louisville, Louisville, KY USA; 4 Department of Periodontics, Virginia Commonwealth University School of Dentistry, Richmond, VA USA; 5 Comprehensive Cancer Center, University of Virginia, Charlottesville, USA

**Keywords:** Periodontitis, Mechanisms of disease

## Abstract

The increasing aging population and aging-associated diseases have become a global issue for decades. People over 65 show an increased prevalence and greater severity of periodontitis, which poses threats to overall health. Studies have demonstrated a significant association between aging and the dysfunction of neutrophils, critical cells in the early stages of periodontitis, and their crosstalk with macrophages and T and B lymphocytes to establish the periodontal lesion. Neutrophils differentiate and mature in the bone marrow before entering the circulation; during an infection, they are recruited to infected tissues guided by the signal from chemokines and cytokines to eliminate invading pathogens. Neutrophils are crucial in maintaining a balanced response between host and microbes to prevent periodontal diseases in periodontal tissues. The impacts of aging on neutrophils’ chemotaxis, anti-microbial function, cell activation, and lifespan result in impaired neutrophil functions and excessive neutrophil activation, which could influence periodontitis course. We summarize the roles of neutrophils in periodontal diseases and the aging-related impacts on neutrophil functional responses. We also explore the underlying mechanisms that can contribute to periodontitis manifestation in aging. This review could help us better understand the pathogenesis of periodontitis, which could offer novel therapeutic targets for periodontitis.

## Introduction

Almost all countries are seeing an increase in the percentage in the population of 65 years of age and older. In 2030, the rate of older adults in the United States is projected to be 21%. Furthermore, the number of persons 85 years and older is projected to increase more than three times between 2014 and 2060, from 6 to 20 million. The aging population has common chronic conditions that share several risk factors and risk indicators with periodontitis (PD), making them more susceptible to PD.^1^ PD is a dysregulated dysbiotic inflammatory disease characterized by long-term inflammation of the periodontium, comprised of gingiva, periodontal ligament, and alveolar bone, with loss of the latter. The primary etiology of PD is essentially pathogenic microorganisms in the dental plaque.^2^ PD is preceded by gingivitis, and it displays clinical signs of red, swollen, and bleeding gingiva. Throughout this stage, pain is not frequently reported.^3^ Gingivitis is reversible; however, it can develop into PD if untreated.^4^ PD can present as mild attachment loss in the early stage, leading to significant damage to the attachment apparatus. In its more severe forms, tooth loss and masticatory function impairment are also present.^5^

PD poses a severe risk to an individual’s overall health and is associated with many systemic diseases, especially aging-related diseases such as cardiovascular disease, Type 2 diabetes (T2D) and Alzheimer’s disease (AD).^6,7^ However, the mechanism of aging-associated PD remains unclear.^8^ Older individuals, especially individuals over 65 years old, have higher expression levels of pro-inflammatory factors in their circulation than younger individuals, causing an increased prevalence and greater severity of PD.^9^ Unfortunately, the dysregulated inflammatory response elicited by the host to combat the dysbiotic microbial community contributes to disease progression. Additionally, accumulated gingival damage due to lack of periodontal regeneration can also worsen PD with aging. Studies indicated that aging individuals’ pro-inflammatory phenotypes of macrophages and T cells may contribute to PD.^1^ On the other hand, previous studies have indicated a close association between neutrophils and the onset and development of PD. Both deficiencies and excessive neutrophil reactions may contribute to PD.^10,11^ Neutrophil migration is impaired in old mice, indicating that aging could dysregulate the neutrophil effector functions, jeopardizing the cell’s role in maintaining tissue homeostasis and preventing PD progression.^12,13^ Furthermore, recent studies claim elderly individuals have a higher ratio of the immunosuppressive subset of neutrophils.^14–17^ Answering the question of how biological aging affects neutrophils’ functional responses can help us better understand the role of this phagocytic cell in the pathogenesis and progression of PD.

This review summarizes the functional mechanism of neutrophils in PD and further discusses how aging leads to neutrophil defects related to PD pathology.

## Neutrophils in health and periodontitis

Neutrophils are the earliest responders to combat invading pathogenic bacteria. Neutrophils differentiate and mature in the bone marrow and are released into circulation. In PD, they migrate to periodontal tissues to respond to the pathogenic bacteria by crosstalking with macrophages and T and B lymphocytes.^18^ The differentiation, maturation, and chemotaxis of neutrophils are strictly regulated to maintain a healthy neutrophil homeostasis, which is crucial for neutrophils to defend against microbial invasion in PD.^19^ In this section, we will review neutrophil homeostasis in healthy conditions, their function in PD, and how neutrophil dysfunction and overreaction may lead to PD.

### Neutrophil differentiation, maturation, and kinetics

**Fig. 1:**
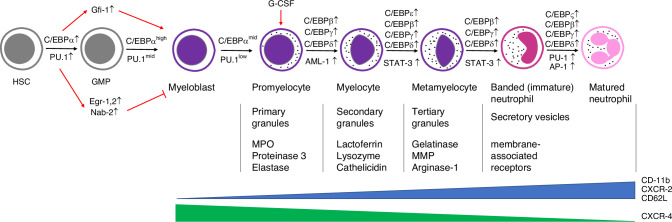
Differentiation and maturation of human neutrophils. In the bone marrow, hematopoietic stem cells (HSC) simultaneously expressing high levels of C/EBPα and PU.1 differentiate into GMP. The downstream regulator of C/EBPα, Gfi-1, promotes the differentiation into neutrophils, while the downstream regulators of PU.1, Egr-1, 2, and Nab-2, inhibit the differentiation into neutrophils. Their interactions result in the differentiation of GMP with high expression of C/EBPα and low expression of PU.1 into myeloblast and promyelocytes. Under the regulation of other members of the C/EBP family and regulators such as AML-1 and STAT-3, promyelocytes differentiate into myelocytes, metamyelocytes, and banded (immature) neutrophils. Regulated by C/EBPζ and AP-1 expression, banded neutrophils differentiate into matured neutrophils. During the differentiation and maturation of neutrophils, the expression of cell surface markers CD-11b, CXCR-2, and CD62L increases, while the expression of CXCR-4 gradually decreases. Primary granules, secondary granules, tertiary granules, and secretory vesicles are produced at different stages of differentiation, starting from promyelocytes. HSC hematopoietic stem cell, GMP granulocyte-macrophage progenitor, G-CSF granulocyte colony-stimulating factor, MPO myeloperoxidase, MMP matrix metalloproteinases

Neutrophils are the first responders in the innate immune response to acute inflammation and pathogen invasion, protecting the host’s systemic health. They can enter the circulation from the bone marrow and migrate into tissues to eliminate pathogens and assist in adaptive immunity.^20^ Under normal physiological conditions, the percentage of neutrophils within the leukocyte population in the peripheral blood is 10–25% in mice and 50–70% in humans.^21^ As depicted in Fig. 1, various molecules are involved in regulating neutrophil differentiation and maturation.

The differentiation of neutrophils starts with granulocyte-monocyte progenitor cells (GMP), which are sourced from hematopoietic stem cells (HSC) in the bone marrow and promoted by C/EBPalpha and PU.1.^22,23^ Laslo et al. first indicated the cross-antagonism between PU.1 and C/EBPalpha.^24^ Although the primary regulators PU.1 and C/EBPalpha promote differentiation in neutrophils and other cell types, the secondary regulator Gfi-1 stimulated by C/EBPalpha only promotes the differentiation to neutrophils. Alternatively, the secondary regulators Egr-1, 2, and Nab-2, which PU.1 stimulates, suppress the differentiation to neutrophils and mutually inhibit each other along with Gfi-1. As a result, cells with higher expression of C/EBPalpha and lower expression of PU.1 differentiate into the myeloblast and further differentiate into neutrophil promyelocyte, the first determined neutrophil.^25,26^ Cells then mature into myelocyte, metamyelocyte, banded neutrophils, and mature neutrophils mediated by the C/EBP family, PU.1, Gfi-1, AML-1, STAT-3, and other regulators.^27,28^ In humans, the term *neutropoiesis* is used to describe the neutrophil ontogeny instead of granulopoiesis since the current evidence indicates that eosinophils, basophils, and neutrophils undergo independent pathways during their maturation starting from HSCs stages.^29,30^ Based on the cluster of differentiation markers CD64+ CD115− cells and with variation in the expression of CD34, CD45RA, or both, four neutrophil-committed progenitors (NCPs) have been identified, defined, and characterized.^31,32^

During maturation, neutrophils increase the expression of CXCR2 and decrease the expression of CXCR4. Chemokines such as CXCL1 (also called KC) and CXCL2 (also called MIP-2), which are secreted by endothelial cells and megakaryocytes outside of the bone marrow, activate CXCR2 signaling, which promotes neutrophil release into the circulation.^27,33^ In contrast, CXCL12 (also called SDF-1) secreted by osteoblasts and other bone marrow stromal cells acts on CXCR4 signaling to retain neutrophils in the bone marrow.^27,33,34^ The decreased expression of CXCR4 during the maturation process ensures that mature neutrophils can be released into the circulation. Activation of CXCR2 signaling in neutrophils allows them to migrate through the vascular barrier and be released into the blood.^35^

Under homeostasis, only matured neutrophils are released into the blood, and the release process is strictly regulated.^36^ Matured neutrophils are released to circulation by decreasing the CXCL12/CXCR4 signal and a high CXCR2 expression level.^37,38^ An investigation indicated that only about 1% of the whole neutrophils were released into the blood in mice under normal conditions.^39^ A circadian rhythm for neutrophil release was reported in a mouse model in which the levels of CXCL12 were highest at ZT21 and lowest at ZT9, indicating indirect regulation by clock signals (Bmal-1, Per1, Per2) from the central nervous system.^40^ This results in the highest level of circulation neutrophils at the light phase (resting phase for mice) and the lowest level at the dark phase (active phase for mice).^40,41^ After being released into the circulation, neutrophils switch to another circadian rhythm by the internal clock. In circulation, neutrophils start to express CXCL2, driven by Bmal1. CXCL2/CXCR2 signal stimulates the process of neutrophil aging, which is suppressed by CXCR4.^38,42^ Fresh-released neutrophils exhibited a CXCR2^high^, CXCR4^low^, and CD62L^high^ phenotype. Aging leads to decreased CXCR2 and CD62L expression and increased expression of CXCR4. This results in degranulation, loss of microvilli, and reduced NET formation and migration ability.^41–44^ Aged neutrophils return to the bone marrow or transmigrate to peripheral tissues under the influence of the CXCL12/CXCR4 signaling, and the apoptotic neutrophils are cleared by macrophage or dendritic cells (DC).^45–48^ After clearing apoptotic neutrophils, macrophages, and DC downregulate the expression of IL-23. The reduction of IL-23 is reported to inhibit the expression of IL-17, granulocyte colony-stimulating factor (G-CSF), and the production of neutrophils, suggesting a feedback mechanism via phagocytosed apoptotic neutrophils for regulating neutrophil homeostasis, a term known as neutrostat.^49^ Although there is still an undefined part about the duration of neutrophils in circulation, most studies indicate that under steady-state conditions, their lifespan is less than one day.^50,51^

### Migration and bactericidal ability of neutrophils in periodontitis

Neutrophils exhibit different patterns of differentiation and kinetics under inflammatory conditions regulated by G-CSF, a cytokine typically produced by macrophages, endothelial cells, and fibroblasts, under lipopolysaccharide (LPS) stimulation.^52,53^ LPS are bacterial surface glycolipids present in the outer membrane of most Gram-negative bacteria.^54^ Specific Gram-negative anaerobic bacteria are enriched in periodontal pockets of patients with PD and are considered a dominant pathogen causing inflammatory bone resorption in PD.^55,56^ G-CSF interacts with the G-CSF receptor and promotes neutrophil development.^25,57^ PU.1 and C/EBPalpha were also reported as crucial transcription factor regulators of the expression of the G-CSF receptor. These two proteins bind specifically to the receptor’s promoter region and the absence of these two binding sites results in a significant decrease in receptor expression.^58^ G-CSF increases neutrophil production and enhances neutrophil adhesion, which is important for neutrophil trafficking during inflammation.^59^ Several studies reported that G-CSF stimulation enhanced the expression of CXCL1 and CXCL2 and suppressed the CXCL12/CXCR4 axis to mobilize the neutrophils to circulation acutely.^60,61^ These studies demonstrate that G-CSF affects neutrophil production, release, and mobilization, making it a critical activating factor for neutrophils.

**Fig. 2:**
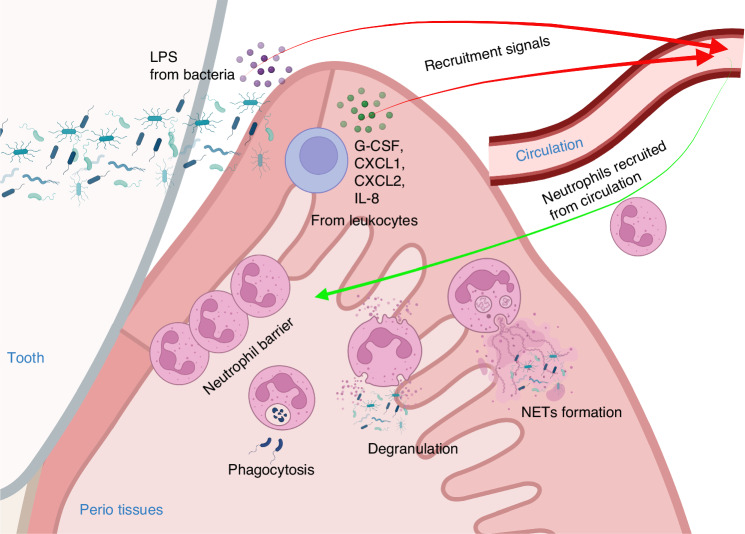
Neutrophil recruitment and antibacterial function in perio tissues. Circulating neutrophils respond to LPS released by periodontal bacterial biofilm, as well as G-CSF, CXCL1, CXCL2, IL-8, and other signals released by residency immune cells. Neutrophils are recruited to perio tissues and form a barrier to defend against bacteria. Recruited Neutrophils can recognize and eliminate bacteria through phagocytosis, degranulation, and NETs formation, thereby maintaining the balance of the periodontal microbiome. Created in https://BioRender.com

As depicted in Fig. 2, Neutrophils in circulation are recruited to the periodontal tissues to protect them from the bacterial biofilm. Chemotactic signals for neutrophil recruitment include LPS, IL-8, CXCL1/2 interacting with CXCR2, and CXCL12/CXCR4 axis, released by the bacteria, periodontal tissues, and residency immune cells.^10,62–67^ Neutrophils are recruited to the periodontal tissues even without oral microbiota in germ-free mice, suggesting immune surveillance of bacterial colonization.^19,68^ The disruption of the balance between the bacteria biofilm and the antibacterial function of neutrophils that protect the host from the bacteria biofilm is a primary cause of the pathogenesis of PD. Neutrophils in circulation respond to chemotactic signals and are recruited into the periodontium, passing through the endothelial cells. The recruitment process of neutrophils to the periodontium is crucial for preventing the invasion of periodontal bacteria. To overcome this barrier, periodontal pathogens such as *Porphyromonas gingivalis* (*P. gingivalis*) secretes a virulence factor, a serine phosphatase enzyme called SerB, which inhibits the secretion of IL-8 from gingival epithelial cells, thereby disrupting the recruitment of neutrophils.^69^ Neutrophils recruited to the periodontal tissues form a barrier between the microbial biofilm of PD-causing pathogens and the gingiva, protecting the periodontal tissues from bacteria invasion.^10^ On the other hand, impaired neutrophil recruitment leads to the overgrowth of pathogenic bacteria and the progression of PD.^19,70^

Neutrophils that enter the inflamed site recognize periodontal bacteria and activate phagocytosis through Fcγ class receptors, complement receptors, and non-phagocytic Toll-Like receptors (TLRs).^71^ In neutrophils, the phagosome maturation process takes place through the fusion of primary and secondary granules to the bacteria-containing phagosome with activation of the NADPH oxidase and the generation of reactive oxygen species (ROS) and hypochlorous acid (HOCl).^72^ On the other hand, neutrophils can also kill extracellular bacteria by releasing their granule content through exocytosis, activating the NADPH oxidase at the neutrophil plasma membrane, and releasing ROS into the tissues.^73^ Neutrophil phagocytosis is a potent weapon against periodontal pathogens and is essential for maintaining the balance of oral microbiota. Previous research indicates that *P. gingivalis* can be phagocytosed and eliminated by neutrophils while also inducing the production of ROS in neutrophils.^74^ On the other hand, gingipain K produced by *P. gingivalis* cleaves IgG1 and IgG3, effectively inhibiting neutrophil opsonin-dependent phagocytic function, resulting in the progression of PD.^75^ Meanwhile, another study has demonstrated that P. *gingivalis* can prevent phagosome maturation and phagocytosis by the crosstalk between the TLR2/1 and C5a receptors.^76^ Clinical research has observed that the phagocytic ability of neutrophils in patients with PD is reduced, especially in rapidly progressive PD, increasing the susceptibility to the disease.^77–80^ These results suggested that neutrophils’ phagocytic mechanism against *P. gingivalis* is not perfect. Neutrophil killing of *P. gingivalis* may require cooperation with other mechanisms, and different strains of *P. gingivalis* may exhibit varying resistance to phagocytosis.

Another strategy neutrophils use to kill pathogens is mobilizing their granules to release their antimicrobial content either to the bacteria-containing phagosome or to the extracellular space through a process known as degranulation. Neutrophil granules are formed during their differentiation process in the bone marrow. Primary or azurophilic granules are formed at the early stage of neutrophil differentiation and contain potent antibacterial proteins such as α-defensins, myeloperoxidase (MPO), proteinase 3, and neutrophil elastase (NE), to name a few.^81^ Secondary or specific granules contain lactoferrin, lysozyme, and pre-cathelicidin; tertiary or gelatinase granules contain various matrix metalloproteinases (MMPs) such as MMP-8 and MMP-9.^82^ Additionally, secretory vesicles are formed by endocytosis and are the latest formed during the neutrophil ontogeny process. Secretory vesicles are enriched in surface membrane-associated receptors such as CD11b/CD18 and CD16. They are necessary for the early stages of the inflammatory response, such as firm adhesion to the endothelial layer.^81,83^ During an infection, primary and secondary granules are primarily targeted to fuse with the bacteria-containing phagosome and release their granule content within the vacuole to minimize damage to host tissues.^84^ However, pathogens developed strategies to evade neutrophil-killing.^84^ Studies characterizing the interaction between human neutrophils and *Aggregatibacter actinomycetemcomitans* (*A. actinomycetemcomitans*), a periodontal pathogen associated with PD, show that this organism induces the exocytosis of azurophilic granules, which contain epinephrine, a catecholamine used by the bacterium to grow.^85^ Furthermore, leukotoxin A, a potent virulence factor of *A. actinomycetemcomitans*, promotes neutrophil degranulation and contributes to PD pathology.^86^ Additionally, studies between neutrophils and *Filifactor alocis*, an emerging periodontal pathogen, show that *F. alocis* induces the activation of the p38 MAPK pathway and the release of secondary granules and secretory vesicles via TLR2 recognition.^87^ Studies have shown high levels of markers associated with neutrophil degranulation, such as MPO and MMP-9, in the oral cavity of patients with PD compared to healthy individuals, indicating that neutrophils from patients with PD display an activated phenotype.^88^

To clear pathogens, neutrophils also rely on forming neutrophil extracellular traps (NETs). Neutrophils undergoing NETosis release a web of decondensed DNA and histones decorated with antimicrobial peptides and neutrophil proteases, which are toxic to microbes. It has been described that when ROS production is required for NET formation, that process results in the release of MPO and NE from primary granules, initiating the formation of NETs.^89^ MPO and NE translocate to the cell nucleus, where they act together to promote chromatin decondensation. Furthermore, post-translational modifications occur to histones where arginine residues are converted to citrulline by protein-arginine deiminase type 4 (PAD-4), and antibodies against these different markers are used to identify NETs by microscopy.^90^ It is worth noting that the ROS produced by mitochondria can also stimulate this process without the assistance of NADPH oxidase.^91^ During NETosis, neutrophils’ proteases activate Gasdermin D (GSDMD) to form membrane pores.^92^ Subsequently, the nuclear envelope and granular membranes disintegrate, allowing the cytoplasmic and NETs to mix. Ultimately, the cell membrane breaks, leading to the release of NETs.^93^ The NET components contain DNA, Histones, and NE, which can capture and destroy pathogens.^89,93^ The production of NETs was initially believed to be invariably associated with the cell death of neutrophils, so this process was termed NETosis.^93,94^ However, an increasing number of studies suggest alternative ways of NET production that can bypass cell death. For example, studies have shown that under the induction of *S. aureus*, neutrophils can rapidly release NETs in 5–60 min, whereas the conventional release takes three to four hours.^95,96^ These cells retain phagocytic activity without a cell nucleus. Alternatively, S Yousefi et al. showed that under complement factor 5a (c5a) stimulation, neutrophils can form NETs by releasing mitochondrial DNA to avoid cell death.^97^ These findings suggest that different stimulatory conditions might have distinct activation pathways for neutrophil NET formation. Although numerous NETs stimulants, including LPS (interacts with TLR4),^98^ phorbol myristate acetate (PMA, interact with protein kinase C),^94^ IL-8, M1 protein,^99^ and direct action of pathogens, have been identified, they have many overlaps with the stimulation of phagocytosis and degranulation. The decisive signaling factors for triggering NET formation remain to be addressed. Recent research has highlighted the competitive relationship between phagocytosis and NET formation, suggesting that the pathogen size detected by dectin-1 might be one of the determining factors.^100^ The antimicrobial functions of NETs against periodontal bacteria are crucial for maintaining oral health. NETs have a lethal effect on various bacteria in the periodontal microbiota.^101^ NETs can effectively capture and eliminate bacteria to prevent their invasion into the gingiva.^102^ However, the killing efficacy against different bacteria is different.^103^ Additionally, patients with papillon–Lefèvre syndrome (PLS), whose neutrophils lack serine proteases that would usually be localized within azurophilic granules, have defects in NETs formation, and these patients suffer from concurrent rapidly progressive PD.^104^

### When neutrophil responses contribute to periodontitis

Defects on those neutrophil antimicrobial functions and homeostasis can lead to oral dysbiosis and initiate/exacerbate PD.^11^ A study on the surface markers of neutrophils in the peripheral blood of patients with PD suggests that PD treatment can reduce the number of suppressive neutrophils (CD16^bright^, CD62L^dim^) which inhibit T-cell proliferation.^105^ Suppressive neutrophils also show decreased adhesion, which affects neutrophil migration and could be crucial for the pathogenesis of PD.^105,106^ Meanwhile, various neutrophil deficiency diseases such as neutropenia, A1AT deficiency, Granulomatous diseases, and others have been confirmed to be associated with PD, emphasizing the importance of neutrophil function in preventing PD.^10^ However, excessive neutrophil responses can exacerbate PD, making neutrophil’s role a double-edged sword in this disease.^10^

As described before, NE is a strong degradative enzyme used against bacteria. On the other hand, it was reported in the literature that the activity of NE is significantly increased in the samples from patients with PD, which may shed light on the cause of tissue destruction seen in PD.^107^ Furthermore, a murine PD model has confirmed that NE’s activity can increase bone loss and disrupt the gingival epithelial cell barrier, leading to bacterial invasion and exacerbating periodontal destruction.^108^ Although the clearance of pathogens by NETs is crucial for suppressing the onset of disease, sustained high concentrations of NETs act as a promoter of PD.^109^ The formation of an oral biofilm attracts neutrophils and stimulates the NET formation.^110^ Meanwhile, the release of NETs in the peripheral blood is significantly elevated in patients with PD.^111^ Several studies have indicated that oral microbiota can neutralize NETs’ antimicrobial activity by degrading their DNA and protein.^112–114^ This results in the persistent production of NETs and the release of harmful proteases, potentially damaging healthy oral tissues and leading to the development of PD.^112–114^ Because of this evidence, NETs have been considered a destructing factor to periodontal tissues, leading to increased susceptibility to pathogen invasion.^109,115^ Recently, research has reported that excessive neutrophil infiltration and NETs formation on periodontal tissue under PD increased the IL-17/Th-17 inflammatory response and triggered periodontal bone loss.^116^ NETs were also reported to worsen Apical PD by activating osteoclasts.^117^ Bone loss and other symptoms were alleviated under DNase I treatment.^116,117^ These studies have demonstrated the double-edged role of NETs in PD. Maintaining a balance of NETs will become a crucial target for treating PD.

It is important to note that macrophages’ clearance of apoptotic neutrophils through a process known as efferocytosis is crucial in sustaining periodontal tissue homeostasis. Neutrophil adhesion and trans-endothelial migration to the tissue are essential steps that regulate neutrophil recruitment to sites of infection. Endothelial locus-1 (DEL-1), a protein secreted by endothelial cells, interacts with neutrophil integrin LFA-1, acting as a gatekeeper of neutrophil extravasation.^118^ A study using DEL-1-deficient mice described massive neutrophilia in the periodontium, which led to increased inflammation and spontaneous alveolar bone loss.^119^ Unlike in circulation, neutrophils recruited in tissue have a delay in their constitutive apoptotic program, which extends their lifespan for a few days to fulfill the antimicrobial mission. A recent study reported that doxorubicin (DOX)-induced neutrophil apoptosis reduced lung inflammation in mice under LPS stress, increased mouse survival rates, and reduced ischemic stroke-induced brain damage.^120^ In PD, *F. alocis*, through its direct interaction with TLR2/6, inhibits neutrophil caspase 3, 8, and 9, key enzymes that regulate neutrophil apoptosis. As a result, *F. alocis* interactions with human neutrophils delay neutrophil apoptosis, prolonging the lifespan of these cells and their functional capacity towards secondary stimuli.^121^ If infected neutrophils don’t display an apoptotic phenotype and remain activated, efferocytic mechanisms at the gingival tissue will not be triggered, which prevents the initiation of the resolution phase of inflammation and fuels periodontal tissue damage.^122^ In summary, a better understanding of how neutrophil functions are regulated in the periodontium would help to define tissue immunity in health and disease.

In recent years, the impact that aging has on neutrophil steady state and functions has garnered significant attention. Aging alters neutrophil homeostasis, further reduces the chemotaxis and antimicrobial function of neutrophils, and extends the duration of inflammatory responses.^16^ Therefore, it is plausible that the alternation of neutrophil functions induced by aging can influence PD in older populations.

## Aging-related alternation of neutrophil functions influences periodontitis

**Fig. 3:**
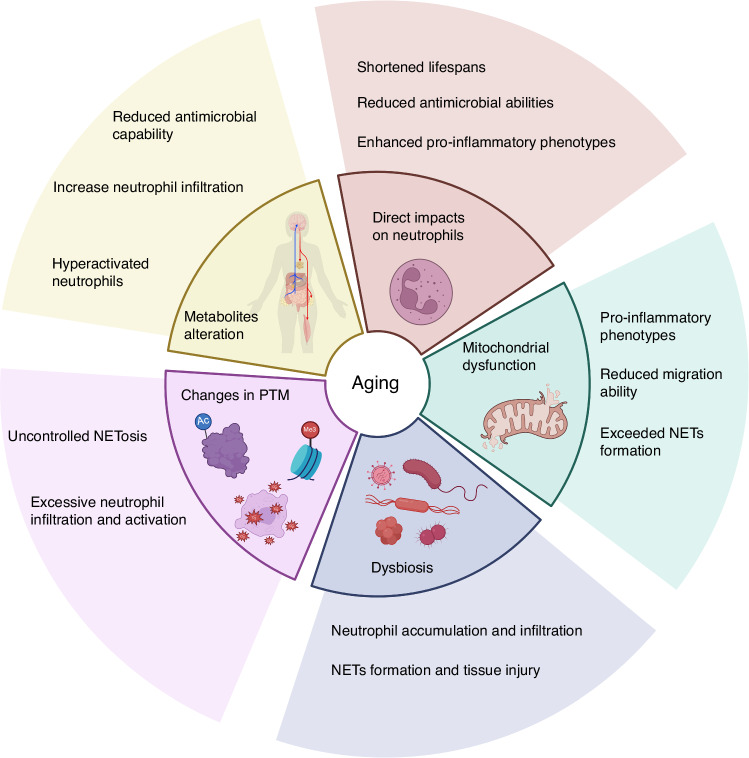
The effects of aging-induced changes on neutrophil functions. Aging can directly impact neutrophils, including shortened lifespan, reduced bactericidal activity, and pro-inflammatory phenotype. Aging induces mitochondrial dysfunction, which may cause reduced neutrophil migration ability, enhanced inflammatory phenotype, and increased NETs formation. Another aging phenotype, dysbiosis, is associated with excessive neutrophil accumulation, which may lead to excessive NETs formation and tissue damage. The impact of aging on PTMs, such as increased histone citrullination and protein oxidation, may lead to excessive neutrophil activation and uncontrolled NETosis. Aging leads to changes in metabolites, resulting in reduced bactericidal activity and hyperactivation of neutrophils. Created in https://BioRender.com

Aging is a continuous decline in biological functions observed at the whole body, organ, tissue, and cellular levels. The deterioration of body functions disrupts homeostasis, leading to mitochondrial dysfunction, DNA damage accumulation, and dysbiosis. It also increases the risk of age-related diseases such as AD, cardiovascular disease, T2D, nonalcoholic fatty liver disease (NAFLD), cancer, and chronic inflammation, including PD.^123–125^ Furthermore, aged individuals exhibit elevated inflammatory markers in their blood and tissues and show a pro-inflammatory status.^126^ It is important to mention that the rate of PD is higher in elderly individuals than in younger individuals, making age a critical determinant of the clinical presentation of PD and a significant focus in PD studies.^127,128^ As we reviewed in the previous section, imbalanced neutrophil functions are closely associated with PD. Therefore, understanding the relationship between aging, neutrophils, and PD becomes crucial. This section will focus on how these aging-induced changes affect neutrophil’s functional responses and can further influence PD (Fig. 3).

### The impact of aging on neutrophil homeostasis and functions

Numerous studies have shown that aging directly affects the homeostasis and function of neutrophils.^16^ The studies indicate that the functionality of HSCs is compromised in aging mice, leading to a reduced capacity to produce mature blood cells, along with increased myeloid potential and decreased lymphoid potential.^129,130^ However, there is no consensus on how the number and the differentiation of neutrophils change in aging individuals. A recent study showed that the proportion of neutrophils in human peripheral blood increases with age, but there was no statistically significant difference between the adult and elderly groups.^131^ Another study reported that the number of neutrophils in the bone marrow of elderly individuals remains unchanged. Still, the response to G-CSF is reduced, which may lead to insufficient neutrophil proliferation during inflammation.^132^ Furthermore, a study comparing the number of neutrophils in healthy young and elderly individuals revealed no difference in the baseline neutrophil counts. Still, there was a significant decline in the ability to release neutrophils from the bone marrow into the blood in elderly individuals.^133^ On the other hand, it was reported that the number of neutrophils in elderly mice increased.^134^ These studies suggest that while the impact of aging on the number of neutrophils remains unclear, aging does affect the release and mobilization of neutrophils. Recent research indicates that in the experimental middle cerebral artery (MCA) model, aged mice exhibit a CD62L^lo^ pro-inflammatory and pro-thrombotic phenotype or CD101^lo^ immature neutrophils after stroke.^135^ Multiple studies have indicated decreased neutrophil chemotaxis in elderly individuals and aged mice.^136,137^ On the other hand, neutrophils under aging conditions have been confirmed to be excessively recruited to tissues.^16,138^ These paradoxical observations may reflect the reduced efficiency and accuracy of neutrophil migration with aging.^139^

Aging also impacts the functionality of neutrophils. Neutrophils from elderly individuals showed a high ratio of early apoptosis, and the increase in neutrophil apoptosis within the periodontal tissue may cause further inflammatory propagation and the inability of these cells to perform antimicrobial functions.^1^ Apoptotic neutrophils lose receptor-dependent inflammatory responses and lack phagocytosis, degranulation, and ROS production functions, which are associated with a weakened immune system in older adults.^140^ Simultaneously, as individuals age, neutrophils’ antimicrobial properties decrease due to reduced phagocytosis and NET formation, leading to increased susceptibility to pathogens in periodontal tissue. A study using TNFalpha-primed human neutrophils indicated that in the sample from elderly individuals, the expression of the IL-8 receptor CXCR2 and the LPS receptor TLR4 in neutrophils is no different from that in young individuals. However, ROS production and NET formation induced by IL-8 and LPS are reduced, suggesting decreased sensitivity to these stimuli.^141^ In contrast, research indicates an increased spontaneous ROS production by neutrophils in elderly individuals related to chronic inflammation,^142^ and ROS production is essential for NETs formation and NETosis.^93^ Furthermore, a recent study has shown that unstimulated neutrophils from aged female mice have higher NETosis inducibility than those from young mice.^143^ Another research using human samples showed that neutrophils from elderly individuals produce more NETs than those from younger people, but their bactericidal ability is weaker.^144^ These results suggested that the enhanced inflammatory phenotypes and various diseases associated with aging may lead to the overactivation of neutrophils and excessive NETs formation.^16,145^ A study targeting individuals over 65 revealed that overreactive neutrophils are detrimental to elderly individuals. The study revealed that groups with a higher neutrophil count showed a significantly lower survival rate.^146^ Although the studies reached different conclusions regarding ROS and NET formation in neutrophils of aged individuals because of the different experimental conditions, most studies pointed out the diminished bactericidal ability of neutrophils in older people. Indeed, a study comparing the phagocytic ability, ROS production, and bactericidal efficiency in neutrophils of elderly and young individuals pointed out that all these three capabilities, as well as chemotaxis in elderly neutrophils, are decreased.^147^

Neutrophils are excessively recruited into tissues despite decreased neutrophil chemotaxis in aging. These neutrophils exhibit shortened lifespans and reduced antimicrobial abilities yet show enhanced pro-inflammatory phenotypes such as increased ROS generation and NETs formation. These results seem contradictory, which may be attributed to varying experimental conditions and accentuate the need for more comprehensive research to understand the impact of aging on neutrophil functional responses. However, it is also plausible that deficiencies in bactericidal function and impaired chemotaxis lead to a compensatory and sustained recruitment of pro-inflammatory neutrophils to the tissues. It can be inferred that during aging, the host may try to compensate for the weakened neutrophil function with excessive neutrophil numbers and activation.

These functional deficiencies and excessive neutrophil responses caused by aging may lead to PD, as we reviewed in the section “When neutrophil responses contribute to periodontitis”. Next, we will discuss some underlying mechanisms.

### Aging-induced mitochondrial dysfunction in neutrophils

Mitochondrial dysfunction has become one of the significant markers of aging.^148^ Aging leads to a decline in mitochondrial respiratory function and ATP production, while the generation of ROS significantly increases.^149^ As described before, mitochondria are involved in all neutrophil bactericidal functions and migration. Several researchers showed that inhibiting mitochondrial ROS production resulted in less activation of degranulation and bactericidal ability movement.^150,151^ In neutrophil migration, intracellular ROS can downregulate actin polymerization to slower neutrophil movement.^152^ Many studies have shown that inhibiting mitochondrial complex I or complex III, as well as the absence of mitochondrial superoxide dismutase 1 and 2 (Sod1 and 2), increases intracellular ROS and significantly reduces neutrophil mobility.^153^ Conversely, increased ROS from dysfunctional mitochondria can activate the NF-κB signaling pathway to promote the inflammatory response.^126^ The role of ROS in the activation of NF-κB in neutrophils under LPS stimulation remains a subject of debate^154,155^. Many studies have indicated that ROS could activate the NF-κB signaling pathway in neutrophils and trigger NETosis, which leads to tissue damage.^156^ Aging-induced mitochondrial dysfunction has multiple effects on neutrophil disorders and could be a potential target for PD treatment. Mitochondrial dysfunction also inevitably leads to changes in metabolites, and the impact of altered metabolites on neutrophils is further discussed in the section “Impacts on neutrophils by age-related alterations in metabolites”.

### Aging-related dysbiosis on neutrophils

Microbial dysbiosis is another hallmark of aging that has been demonstrated to be associated with a reduction in beneficial gut microbiota.^126^ The gut and oral mucosa are the two largest microbial communities in the body. A series of recent studies using healthy elderly individuals have indicated that periodontal dysbiosis is associated with cerebrospinal fluid biomarkers and brain Aβ load^157,158^ related to AD. Additionally, *P. gingivalis*, a keystone pathogen associated with PD, is found in the brain tissue samples from AD patients.^159^ Furthermore, several studies have suggested that *P. gingivalis* can invade the brain through the peripheral nerves or blood-brain barrier.^160,161^ Alternatively, gut dysbiosis has also been identified to be strongly associated with AD, establishing an oral gut-brain axis.^162^ In the section “When neutrophil responses contribute to periodontitis”, we discussed the relationship between neutrophils and oral dysbiosis. Unlike in the oral, neutrophils in the gut do not directly interact with the gut microbiota. This is due to the intestinal epithelial barrier isolating commensal bacteria and blocking their potential toxic signals. However, the situation changes when this barrier is compromised due to aging or other factors.^125^ It has been reported that gut dysbiosis induced by aging + calorie-dense obesogenic diet increases neutrophil number and swarming.^163^ Moreover, gut dysbiosis can decrease gut microbial diversity and changes in microbial-derived metabolites.^164^ These metabolites play an essential role in regulating neutrophil functional responses. For example, indoles, derived from the tryptophan metabolism, can protect normal tissues from intestinal inflammation by inhibiting the activity of MPO.^165^ Research showed that antibiotics-induced gut dysbiosis leads to reduced synthesis of acetate, an immune-regulating metabolite, resulting in neutrophil accumulation and tissue injury.^166^ Another article also discusses acetate, showing that under *Clostridium difficile* infection after antibiotic-induced dysbiosis, acetate acts as a neutrophil activator to increase the recruitment and antibacterial function via free fatty acid receptor 2 (FFAR2).^167^ Recent research demonstrated that gut dysbiosis, especially the reduced abundance of *R. intestinalis*, results in the lack of butyrate, a metabolite produced by *R. intestinalis*. This leads to neutrophil infiltration and NET formation, resulting in an abdominal aortic aneurysm (AAA) onset and development.^168^ Although it is known that these microbiome metabolites are crucial in regulating neutrophil function, there is still a lack of comprehensive research to uncover whether these microbiome metabolites can serve as regulators that could mediate neutrophil homeostasis and functions in other locations, such as in the periodontium. Directly mediating aging-induced dysbiosis on oral neutrophils can potentially become a new research direction in PD.

### Changes in post-translational modifications during aging affect neutrophil responses

The impact of aging on different proteins’ post-translational modifications (PTMs) is becoming a popular topic. PTM processes such as phosphorylation, methylation, acetylation, ubiquitination, and oxidation are dynamically regulated by specific modifying enzymes whose activities require metabolites that either serve as co-substrates or act as activators/inhibitors. Emerging evidence reveals that the PTMs of histones are essential in regulating neutrophil death. For example, histone citrullination promotes a rapid NET formation independent of NADPH oxidase (NOX) and calcium influx.^169^ While the impact of aging on histone citrullination requires further investigation, a study suggests that aging is associated with increased citrullination on histones.^170^ Increased histone citrullination induced uncontrolled NET formation and NETosis, potentially contributing to various NET-related diseases such as sepsis, systemic lupus erythematosus (SLE), and cancer progression.^171^ Furthermore, as we discussed earlier, excessive NET production and NETosis can harm periodontal tissues and lead to the deterioration of PD.

Another common PTM regulated by aging is protein oxidation, a modification that can impact the structure and function of proteins and trigger the related pathway. For example, advanced oxidation protein products (AOPPs), which are biomarkers of oxidative stress, are reported to be more abundant in aging adults (age 65–85) than in younger individuals (age 25–45).^172^ Increased levels of AOPP have been demonstrated to impact neutrophil function. Researchers reported that AOPP modified from human serum albumin and collagen triggered neutrophil ROS production, which may induce tissue damage.^173,174^ In addition, the concentration of oxidized low-density lipoprotein (Ox-LDL), an oxidation product of LDL, has been demonstrated to be higher in the blood of elderly individuals and become a biomarker for cardiovascular diseases.^175,176^ Current research suggests that Ox-LDL activates neutrophils to generate NETs, causing damage to vascular endothelial cells and triggering inflammation^177^, which may further lead to PD progression. Oxidation modification can accumulate during aging and impact the structure and function of proteins.^178^ Therefore, critical neutrophil proteins may lose functionality, affecting their activity when oxidation modifies them. Indeed, research indicated that oxidation of the Fc region of Immunoglobulin G (IgG) can decrease the ability to bind to Fc receptors, which is essential to activating neutrophils and other immune cells.^179,180^ However, research on the oxidative modifications of these crucial proteins specific to neutrophils is currently scarce.

Various PTMs, such as acetylation and deacetylation, regulate neutrophils’ activation and function. Acetylation of NF-κB subunit RelA/p65 is essential for the transcriptional function of NF-κB signaling pathway.^181^ During inflammation, NF-κB regulates the production of neutrophils and inhibits apoptosis to sustain the survival of neutrophils.^182^ It also increases the generation of NETs and ROS in neutrophils^183^ and has been demonstrated to have elevated activity in the periodontal tissues of PD.^184^ Sirt1, a nicotinamide adenosine dinucleotide (NAD)-dependent deacetylase can deacetylate RelA/p65, thereby inhibiting the transcriptional activity of NF-κB and disrupting the expression of downstream genes to protect against inflammaging, a condition characterized by proinflammatory phenotypes and declined immune function, and tissue damage caused by NF-κB signaling.^185–187^ The deacetylation of NF-κB by Sirt1 is crucial for inflammation resolution. Studies have confirmed that the loss of Sirt1 during LPS-induced inflammation results in excessive neutrophil infiltration, leading to kidney damage.^188^ Interestingly, studies have demonstrated a decline in the expression of Sirt1 with age in the brains, livers, skeletal muscles, and white adipose tissue of mice, as well as in the brains of rats.^189,190^ Meanwhile, NF-κB activation increased in elderly individuals.^191^ These findings suggested an association between aging, deacetylation of NF-κB, and neutrophil excessive activation, potentially leading to PD. Studies have already noted a correlation between periodontal disease and the decreased expression levels of deacetylases in human saliva or the loss of Sirt1 function.^192,193^ Understanding the relationship between aging, deacetylases, and neutrophil dysregulation could provide new targets for periodontal disease research.

### Impacts on neutrophils by age-related alterations in metabolites

The metabolic pathways, such as glycolysis, citric acid cycle, oxidative phosphorylation, fatty acid, and protein synthesis, form a coordinated network crucial for providing energy and maintaining biological processes. Metabolites are small molecules generated during metabolic processes. They serve as basic materials for energy production and the synthesis of other structures and also play a role in cellular signaling.^194^ The well-known phenotypes of aging include the decline in metabolic functions and the imbalance of metabolic homeostasis,^195^ which may lead to metabolic diseases such as T2D and NAFLD.^123,124^ Moreover, aging also alters the profile of metabolites which may influence immune function.^196^ For example, nicotinamide adenine dinucleotide (NAD+), a coenzyme involved in redox reactions, has declined with aging.^197,198^ The decreased levels of available NAD+ will slow down the rate of glycolysis and ATP production in neutrophils, thereby disrupting the functionality of neutrophils, such as the cell’s ability to form NETs. This has been demonstrated to diminish mice’s antimicrobial capability and increase neutrophil infiltration.^199^ Meanwhile, recent research has found that NAD+ treatment can reduce neutrophil infiltration in various tissues under LPS-induced inflammation in mice, decrease tissue damage, and improve the survival rate of mice.^200^ Researchers have already focused on the potential association between NAD+ and PD. For instance, a study indicated that Sirt6, a NAD+-dependent deacetylase, plays a crucial role in inflammation resolution and was downregulated in diabetic PD.^201^ Furthermore, the expression of another NAD+-dependent deacetylase, Sirt3, decreases with the decline in NAD+ levels and the aging process. This results in oxidative damage to periodontal tissues and age-related PD.^202^ Further investigations into the association between the decline in NAD+ levels during the aging process and the dysregulation of neutrophils may offer new possibilities for treating PD.

Glutamine is another metabolite that decreases with age. Glutamine deficiency can impair autophagic function, and supplementing with glutamine has been shown to prevent oxidative stress-induced cellular damage.^203^ Glutamine can serve as an energy synthesis substrate for neutrophils. In vitro studies indicated that glutamine-treated neutrophils exhibit enhanced antimicrobial capabilities.^204^ Another study showed that glutamine treatment facilitates rapid recovery of peripheral blood neutrophil numbers to baseline levels following the stress response induced by intense exercise.^205^ Furthermore, glutamine was an anti-inflammatory regulator under LPS-induced inflammation in human dental pulp cells by inhibiting the NF-κB pathway.^206^ These results suggest that glutamine may drive the early resolution of periodontal tissue inflammation by enhancing the functionality of neutrophils and their anti-inflammation function.

Glucose and free fatty acids are among the most essential metabolites, and it has been demonstrated that they are present at higher plasma levels in elderly individuals.^207^ Research has indicated that high glucose levels and free fatty acids can induce neutrophil ROS and NET production, resulting in tissue damage.^208,209^ Therefore, diabetes has been confirmed to hyperactivate neutrophils and delay neutrophil apoptosis, causing damage to periodontal tissues.^210^ In addition to these metabolites, numerous other metabolites are regulated by aging.^198^ The impact of these metabolites on neutrophil functions will provide many new targets for PD research.

## Conclusion

**Fig. 4:**
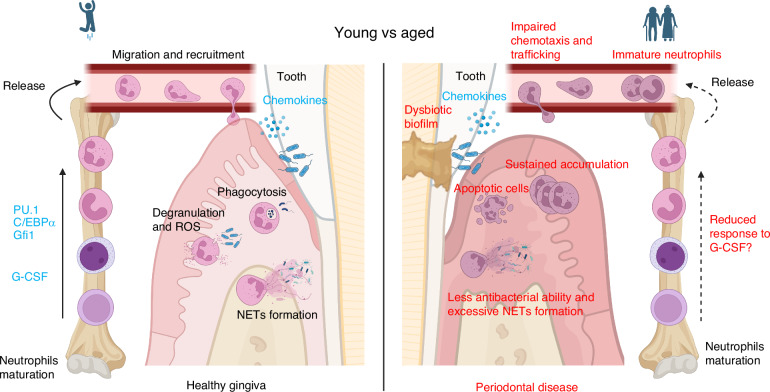
Neutrophils in young and aged conditions contribute to periodontal health/disease. In young individuals, neutrophils differentiate into mature neutrophils in the bone marrow under the regulation of PU.1, C/EBPα, Gfi1, G-CSF, and other regulators. Neutrophils are recruited by chemokines to periodontal tissues from circulation. They defend against microbial invasion to maintain healthy gingival through phagocytosis, degranulation, ROS, and NET formation. Under aging conditions, the differentiation of neutrophils may remain unaffected, but the response to G-CSF is weakened. More immature neutrophils are released into circulation, while the chemotaxis and trafficking of neutrophils show defects. In periodontal tissues, neutrophils exhibit sustained accumulation and increased apoptotic cells. Less antibacterial abilities and excessive NET formation lead to tissue damage, triggering periodontitis. Created in https://BioRender.com

PD is a persistent oral disease that is challenging to control and poses a significant threat to human health. Neutrophils play a crucial role in the periodontium, essential for maintaining the balance between the oral microbiota and the immune system. While neutrophils possess potent bactericidal capabilities, if these functions are dysregulated, they can harm the host tissue. Aging directly disrupts the chemotaxis, recruitment, and bactericidal ability of neutrophils. Biological events contributing to PD are often due to the overreaction and dysfunction of neutrophils that are common in aging (Fig. 4). Age-related impacts on mitochondrial dysfunction, microbial dysbiosis, PTMs, and metabolites are key factors that compromise neutrophil functions but lack comprehensive research. Meanwhile, research on the impact of aging on the differentiation and maturation of neutrophils has inconsistent results. The exact impact of aging on the crosstalk between neutrophils and other immune cells, the cell cycle of neutrophils, and whether these effects might expedite the development of PD remains unclear. Testing the changes in neutrophil responses to cytokines released from other immune cells in aged individuals can potentially develop new therapies that inhibit overactivated neutrophils or increase their bactericidal efficiency to prevent PD. Aging leads to enhanced inflammatory phenotypes that may affect the NETs formation by neutrophils, and excessive NETs formation is one of the important causes of PD. Studying the induction and degradation of NETs in aging models may help find ways to reduce tissue damage caused by excessive NETs formation. Thus, further research into the mechanisms between aging and the functional impairment of neutrophils will provide additional insights to explore the prevention and treatment of PD.
